# Xixin Decoction’s novel mechanism for alleviating Alzheimer’s disease cognitive dysfunction by modulating amyloid-β transport across the blood–brain barrier to reduce neuroinflammation

**DOI:** 10.3389/fphar.2024.1508726

**Published:** 2025-01-06

**Authors:** Chaokai Yang, Enlong Zhao, Hu Zhang, Liqi Duan, Xinyue Han, Hongli Ding, Yan Cheng, Dengkun Wang, Xiaojing Lei, Yongchang Diwu

**Affiliations:** ^1^ The First Clinical Medical College of Shaanxi University of Chinese Medicine, Xianyang, China; ^2^ Key Research Laboratory for Prevention and Treatment of Cerebrospinal diseases, Shaanxi Provincial Administration of Traditional Chinese Medicine, Xianyang, China; ^3^ Discipline Innovation Team for Neurodegenerative Diseases of Shaanxi University of Chinese Medicine, Xianyang, China; ^4^ College of Basic Medicine, Shaanxi University of Chinese Medicine, Xianyang, China

**Keywords:** Alzheimer’s disease, Xixin Decoction, blood-brain barrier, amyloid-beta, neuroinflammation

## Abstract

**Purpose:**

Xixin Decoction (XXD) is a classical formula that has been used to effectively treat dementia for over 300 years. Modern clinical studies have demonstrated its significant therapeutic effects in treating Alzheimer’s disease (AD) without notable adverse reactions. Nevertheless, the specific mechanisms underlying its efficacy remain to be elucidated. This investigation sought to elucidate XXD’s impact on various aspects of AD pathology, including blood-brain barrier (BBB) impairment, neuroinflammatory processes, and amyloid-β (Aβ) deposition, as well as the molecular pathways involved in these effects.

**Methods:**

*In vitro* experiments were conducted using hCMEC/D3 and HBVP cell coculture to establish an *in vitro* blood-brain barrier (BBB) model. BBB damage was induced in this model by 24-h exposure to 1 μg/mL lipopolysaccharide (LPS). After 24, 48, and 72 h of treatment with 10% XXD-medicated serum, the effects of XXD were assessed through Western blotting, RT-PCR, and immunofluorescence techniques. *In vivo*, SAMP8 mice were administered various doses of XXD via gavage for 8 weeks, including high-dose XXD group (H-XXD) at 5.07 g kg^-1^·d^-1^, medium-dose XXD group (M-XXD) at 2.535 g kg^-1^·d^-1^, and low-dose XXD group (L-XXD) at 1.2675 g kg^-1^·d^-1^. Cognitive function was subsequently evaluated using the Morris water maze test. BBB integrity was evaluated using Evans blue staining, and protein expression levels were analyzed via ELISA, Western blotting, and immunofluorescence.

**Results:**

*In vitro* experiments revealed that XXD-containing serum, when cultured for 24, 48, and 72 h, could upregulate the expression of P-gp mRNA and protein, downregulate CB1 protein expression, and upregulate CB2 and Mfsd2a protein expression. *In vivo* studies demonstrated that XXD improved spatial learning and memory abilities in SAMP8 mice, reduced the amount of Evans blue extravasation in brain tissues, modulated the BBB-associated P-gp/ECS axis, RAGE/LRP1 receptor system, as well as MRP2 and Mfsd2a proteins, and decreased the accumulation of Aβ in the brains of SAMP8 mice. Additionally, XXD upregulated the expression of TREM2, downregulated IBA1, TLR1, TLR2, and CMPK2 expression, and reduced the levels of pro-inflammatory factors NLRP3, NF-κB p65, COX-2, TNF-α, and IL-1β in the hippocampal tissues.

**Conclusion:**

XXD may exert its effects by regulating the P-gp/ECS axis, the RAGE/LRP1 receptor system, and the expression of MRP2 and Mfsd2a proteins, thereby modulating the transport function of the BBB to expedite the clearance of Aβ, reduce cerebral Aβ accumulation, and consequently inhibit the activation of microglia induced by Aβ aggregation. This process may suppress the activation of the CMPK2/NLRP3 and TLRs/NF-κB pathways, diminish the production of inflammatory cytokines and chemokines, alleviate neuroinflammation associated with microglia in the brain of AD, and ultimately improve AD pathology.

## 1 Introduction

**FIGURE 1:**
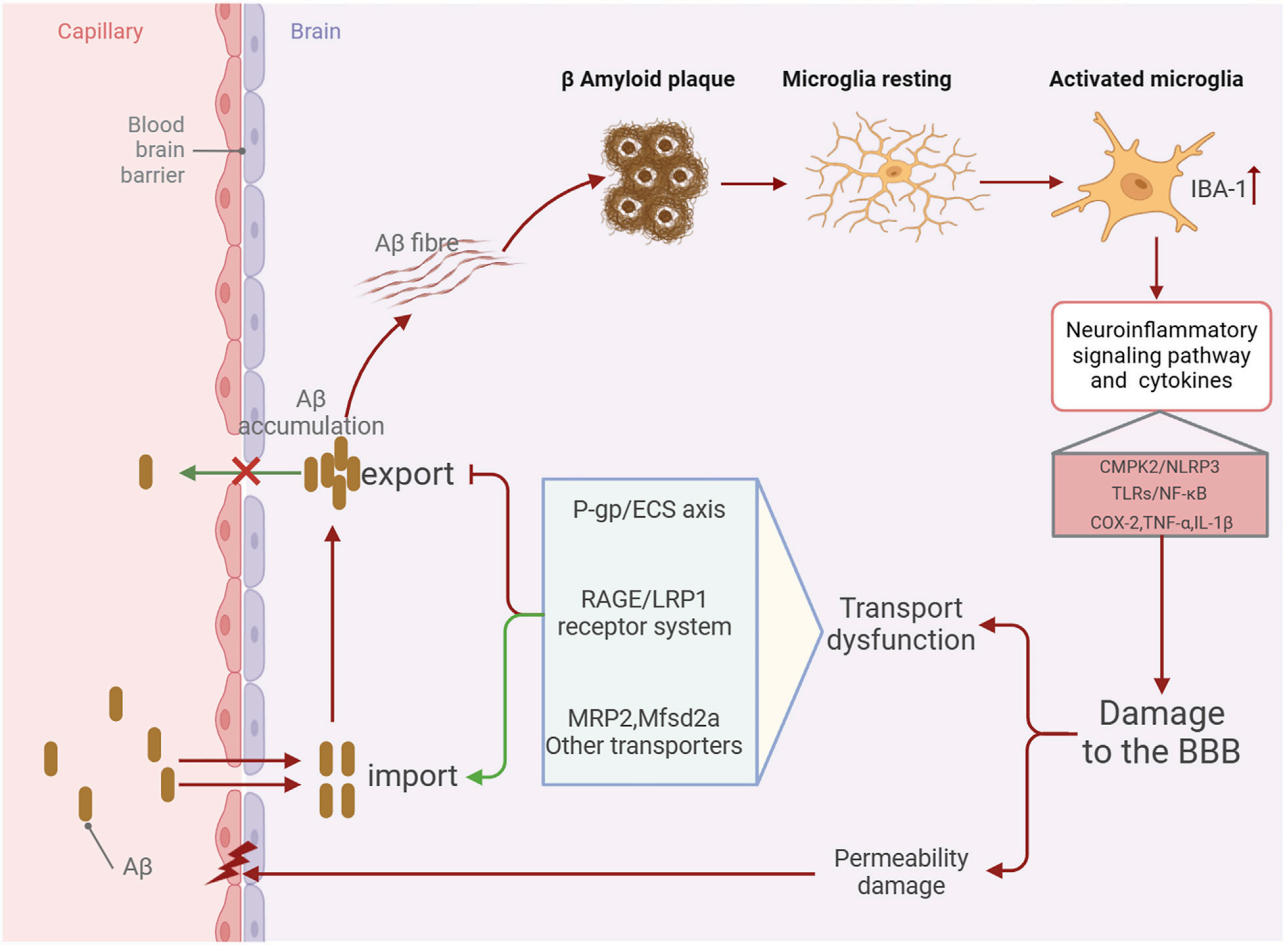
Schematic diagram of the mechanism by which BBB damage in the AD brain aggravates Aβ accumulation and promotes neuroinflammation.

Alzheimer’s disease (AD) is the primary type of dementia among the elderly and ranks as the fifth most common cause of death globally, impacting an estimated 50 million individuals around the world. Owing to the acceleration of population aging, the prevalence of AD is projected to double by the year 2050 (Dumurgier and Sabia, 2020). The blood-brain barrier (BBB) is a complex vascular structure composed of multiple cell types. It isolates the central nervous system (CNS) from the peripheral bloodstream, tightly controlling the movement of molecules and ions to protect the brain from toxins and pathogens and maintain an environment conducive to normal neuronal function (Peng et al., 2021). Currently, the amyloid-β (Aβ) plaque deposition and neurofibrillary tangles resulting from tau protein hyperphosphorylation are considered typical pathological features of AD. The BBB is integral to the clearance of cerebral metabolic waste and is crucial in facilitating Aβ transport across the brain. The typical movement of Aβ across the blood-brain barrier is facilitated by numerous transporters, including LRP1, RAGE, and P-gp (Van Gool et al., 2019; Kim and Priefer, 2020; Vulin et al., 2023; Xing et al., 2023). As Alzheimer’s disease progresses, these transporters show dysregulation in expression and function, resulting in aberrant Aβ transport and deposition (Hanafy et al., 2023; Wang et al., 2021). Neuroinflammation is an inflammatory response in the central nervous system (CNS) triggered by pathological damage in either the periphery or the CNS, leading to the production of proinflammatory cytokines such as interleukin-1beta (IL-1β), IL-6, IL-8, and tumor necrosis factor (TNF), as well as chemokines, complement proteins, and various small molecule messengers, including prostaglandins, nitric oxide (NO), and reactive oxygen species (ROS) (Leng and Edison, 2021). Microglia, the central nervous system’s equivalent of macrophages, are crucial for maintaining CNS homeostasis and are widely regarded as the primary drivers of neuroinflammation in AD (Eshraghi et al., 2021; Chen et al., 2021). In the early stages of AD, activated by Aβ plaque accumulation, microglia produce inflammatory mediators such as cytokines and chemokines, facilitating Aβ clearance and further microglial recruitment (Wang et al., 2023). However, as the disease advances, excessive Aβ accumulation can result in overactivation of microglia, leading to the production of excessive proinflammatory cytokines such as IL-1β, IL-6, and TNF-α. This leads to chronic and sustained neuroinflammation, causing increased neuronal cell death (Huang et al., 2022). Consequently, a vicious cycle is established among Aβ accumulation, activated microglia, and proinflammatory factors (Wei et al., 2022). Simultaneously, neuroinflammation driven by microglial activation further disrupts BBB integrity and transport function (Hussain et al., 2021; Lai et al., 2021; Chaves et al., 2017; Barabási et al., 2023) (Figure 1). Thus, regulating and repairing BBB integrity and transport dysfunction, reducing pathological brain Aβ accumulation, inhibiting microglial activation, and alleviating neuroinflammation may be important approaches to improving AD development.

Presently, five drugs approved by the US Food and Drug Administration (FDA) for treating Alzheimer’s disease include memantine, aducanumab, and three acetylcholinesterase inhibitors (Argueta et al., 2022). These medications only temporarily treat Alzheimer’s symptoms, but fail to control or reverse its underlying pathology (Long et al., 2021). Traditional Chinese medicine (TCM), known for its multi-component, multi-pathway, and multi-target effects, provides unique advantages and significant potential for the prevention and treatment of Alzheimer’s disease (Ding et al., 2022; Chiu et al., 2021; Liu et al., 2024). Based on long-term clinical practice and research of ancient and modern literature, Professor Yongchang Diwu from our research team has innovatively proposed that “marrow deficiency and turbid phlegm” is the pathological foundation for the development of AD, and the traditional famous formula “Xixin Decoction” is chosen for clinical treatment (Diwu, 2016).

Xixin decoction (XXD) originates from the “*Syndrome Differentiation Records”* by Chen Shiduo, a famous Chinese physician in the Qing Dynasty. The seminal work was written circa 1,687, over three centuries ago. XXD has the efficacy of benefiting qi, producing sperm, nourishing marrow, dissipating phlegm for resuscitation, and enhancing intelligence (Wang and Yan, 2009). It is a well-established prescription for dementia that has been clinically verified many times (Xiong et al., 2017; Zeng and Zhou, 2018), and it is also a representative formula for the treatment of dementia with marrow deficiency and turbid phlegm in traditional Chinese medicine (Wang and Yan, 2009). Pharmacological research indicates that XXD and its active metabolites exert anti-AD effects (Yang et al., 2017; Wu et al., 2022; Wang et al., 2022; Hajialyani et al., 2019; Kong et al., 2017).

Furthermore, previous research has found that XXD can affect mouse hippocampal synaptic ultrastructure, protect synaptic integrity (Gao et al., 2018), influence the levels of synaptic function-related proteins, reduce neuronal and synaptic damage, and participate in synaptic plasticity (Lei et al., 2020; Qu et al., 2019). Additionally, evidence indicates that XXD can lower toxic Aβ levels in the brain and promote cholinergic nerve repair (Chen et al., 2020). However, the specific pathways through which XXD affects pathological brain Aβ accumulation and inflammatory toxicity require further research.

Therefore, this study utilized *in vitro* BBB models (hCMEC/D3 and HBVP cell coculture) and SAMP8 mouse models to demonstrate the key role of BBB damage in AD, conduct an in-depth investigation of the relationship between BBB transport dysfunction, neuroinflammation, and pathological brain Aβ accumulation in AD, and reveal the possible pathways involved. Furthermore, XXD-medicated serum and granules were used to intervene in both the *in vitro* BBB models and the animal models to detect relevant indicators to verify whether XXD can regulate and repair BBB integrity and transport dysfunction, reduce pathological brain Aβ accumulation, and inhibit microglia-related neuroinflammation, thereby improving AD. The findings are expected to provide a scientific foundation for the clinical application of XXD.

## 2 Materials and methods

### 2.1 Cells and animals

Immortalized human brain microvascular endothelial cells (hCMEC/D3) and immortalized human brain pericytes (HBVP) were obtained from Shanghai Zhongqiao Xinzhou Biotechnology Co., Ltd. (Shanghai, China).

Male SAMP8 mice (60, SPF grade) and male SAMR1 mice (12, SPF grade), 14 weeks old, weighing (30 ± 5) g, were purchased from Zhishan (Beijing) Health and Medical Research Institute Co., Ltd. (Beijing, China), with animal production license number SCXK (Beijing) 2016-0010. Additionally, 20 male SPF-grade SD rats, 6 weeks old, weighing (200 ± 20) g, were obtained from Chengdu Dashuo Biotechnology Co., Ltd. (Chengdu, China), license number SCXK (Sichuan) 2020-030. All animals were housed in the SPF-grade facility of the Shaanxi Collaborative Innovation Center of Chinese Medicinal Resources Industrialization. They were maintained in individually ventilated cages (IVC) under controlled environmental conditions: 12-h light/dark cycle, ambient temperature of 20°C–25°C, relative humidity of 45%–55%, and noise levels ≤60 dB. Standard laboratory chow and water were provided *ad libitum*. This study was conducted in accordance with the guidelines of the Animal Experimental Ethics Committee of Shaanxi University of Chinese Medicine. All experimental protocols were approved by the university’s Animal Ethics Committee (approval numbers: SUCMDL20220110001 and SUCMDL20211001001).

### 2.2 Antibodies and reagents

Fetal bovine serum (FBS, FBS00321-1) was purchased from AusGeneX (Australia). Sodium pentobarbital (Lot No. 3G223G27) was obtained from Shengxing Biotechnology Co., Ltd. (China). Antibodies against cannabinoid receptor 1 (CB1, AI10227475) and P-glycoprotein (P-gp, BA07303498) were purchased from Bioss (Beijing, China). The antibody against cannabinoid receptor 2 (CB2, 218402) was purchased from Lifespan Biosciences (United States). Rat IL-1β ELISA Kit (Lot No. 20220714-20174A), Rat COX-2 ELISA Kit (Lot No. 20220714-20359A), Rat Aβ_1-42_ ELISA Kit (Lot No. 20220714-20127A), Rat NF-κBp65 ELISA Kit (Lot No. 20220714-21622A), Rat NLRP3 ELISA Kit (Lot No. 20220714-20810A), and Rat TNF-α ELISA Kit (Lot No. 20220714-20852A) were purchased from Enzyme-linked Biotechnology Co., Ltd. (Shanghai, China). Anti-TLR1 antibody (AA07135689), Anti-TLR2 antibody (BA06081077), and Anti-TREM2 antibody (BA08065558) were purchased from Bioss (Beijing, China). The antibody against IBA1 (8525-1904) was purchased from Cell Signaling Technology (CST, United States). Anti-CMPK2 antibody (8525-1904) was purchased from Arigo Biolaboratories Corporation (Taiwan, China). Antibodies against AGER (GR3402053-12) and TLR2 (GR3359428-10) were purchased from Abcam (UK). BCA Protein Assay Kit (P0010) was obtained from Beyotime Biotechnology (Shanghai, China). Anti-Mfsd2a (724A041) and Anti-LRP1 (722A022) antibodies were purchased from Absin Bioscience (Shanghai, China). Anti-MRP2 antibody (BA02187624) was purchased from Bioss (Beijing, China). Anti-GAPDH antibody (AB-PR-001) was obtained from Xianzhi Bioscience (Hangzhou, China). HRP-conjugated goat anti-rabbit IgG secondary antibody (A0208) and anti-fluorescence quenching mounting medium (G1401) were purchased from Beyotime Biotechnology (Shanghai, China). Cy3-conjugated goat anti-rabbit IgG (GB21303) and DAPI (G1012) were purchased from Servicebio (Wuhan, China).

### 2.3 Composition and quantitative analysis of the major metabolites of XXD

The XXD was composed of Panax ginseng C. A. Mey. [Araliaceae; Ginseng Radix et Rhizoma] 30 g, Poria cocos (Schw.) Wolf [Polyporaceae; Poria cocos sclerotium] 30 g, Ziziphus jujuba Mill. [Rhamnaceae; Semen Ziziphi Spinosae] 30 g, Pinellia ternata (Thunb.) Makino [Araceae; Pinelliae ternatae rhizoma] 15 g, Citrus reticulata Blanco [Rutaceae; Citri reticulatae pericarpium] 9 g, Massa Medicata Fermentata [Fungi; Massae Medicatae Fermentatae] 9 g, Aconitum carmichaelii Debx. [Ranunculaceae; Radix Aconiti Lateralis Preparata] 3 g, Acorus tatarinowii Schott [Acoraceae; Rhizoma Acori Tatarinowii] 3 g, and Glycyrrhiza uralensis Fisch. ex DC. [Fabaceae; Radix Glycyrrhizae Preparata] 3 g.

Ten batches of botanical drug decoction were purchased from Beijing Tong Ren Tang Pharmaceutical (Table 1). They were soaked in water at a ratio of 1:10 for 1 h, decocted for an additional hour, and filtered through gauze. Subsequently, the residue was decocted again with eight times the amount of water for 30 min and filtered through gauze. The two filtrates were combined, concentrated to 400 mL, and then freeze-dried into powder.

**TABLE 1:** Information on 10 batches of Xixin Decoction.

Lot	Panax ginseng	Glycyrrhiza uralensis	Citrus reticulata	Ziziphus jujuba	Acorus tatarinowii	Massa medicata fermentata	Poria cocos	Pinellia ternata	Aconitum carmichaelii
S1	22030201	20201202	200601	S20220201	s20220502	202106001	211201	210305	220401
S2	22030202	20201203	200602	S20220202	s20220503	202106002	211202	210304	220402
S3	22030203	20201204	200603	S20220203	s20220504	202106004	211206	210303	20210301
S4	22030204	20201205	200604	S20220401	200901	202106005	211207	210302	20210302
S5	22030205	20201206	200605	S20220402	200904	202106006	211208	210301	20210303
S6	200501	20201207	2022111019	210301	200906	202106009	2203001	210208	210801
S7	200502	220301	2022111018	210302	s20220505	2012001	2203002	210207	210802
S8	200503	220302	2022111020	210303	s20220506	2012002	2203003	210206	210803
S9	200504	220303	2022111017	210304	s20220507	2012003	201001	210205	210804
S10	200505	220304	2022111019	210305	s20220508	2012004	201002	210204	210805

Liquiritin (Lot: Z07J12X136344), spinosin (Lot: N10GB167286), hesperidin (Lot: K09S11L123847), ginsenoside Rg1 (Lot: C27N11Q132589), ginsenoside Re (Lot: J25GB152733), β-asarone (Lot: J23GB155719), ginsenoside Rb1 (Lot: O16GB158610), and ammonium glycyrrhizinate (Lot: M04GB140062) (all were provided by Shanghai yuanye Bio-Technology Co., Ltd., with a mass fraction ≥98%) were used as reference substances for the quantitative determination of the XXD freeze-dried powder. The ginsenosides Rg1, Re, and Rb1 were derived from Radix Ginseng, the hesperidin from Pericarpium Citri Reticulatae, the liquiritin and ammonium glycyrrhizinate from Radix et Rhizoma Glycyrrhizae, the β-asarone from Rhizoma Acori Tatarinowii, and the spinosin from Semen Ziziphi Spinosae. Following precision weighing of the reference substances, they were dissolved in 70% methanol to prepare the solutions. Each solution was injected into an HPLC system with a precisely measured volume of 10 μL. The regression equations were obtained using the reference substance concentration (μg·mL^−1^) as the independent variable and the peak area as the dependent variable (Table 2).

**TABLE 2:** Reference product linear relationship and its range.

Metabolite	Linear relationship	*R* ^2^	Linear range
Liquiritin	Y = 13560x+41.907	0.9998	0.018671875–0.29875
Spinosin	Y = 13492x+54.248	0.9997	0.42–0.013125
Hesperidin	Y = 10433x+63.481	0.9997	0.013219–0.545
Ginsenoside Rg1	Y = 1,421x+30.341	0.9998	4.72–0.07375
Ginsenoside Re	Y = 1822x+24.366	0.9999	2.57–0.020078125
β-asarone	Y = 58627x+170.54	0.9997	0.003125–0.4
Ginsenoside Rb1	Y = 3571.5x+48.668	0.9996	0.013125–1.68
Ammonium glycyrrhizinate	Y = 3380.8x+22.3	0.9998	0.034063–0.545

The XXD freeze-dried powder (1 g) was placed in a stoppered Erlenmeyer flask. Subsequently, 10 mL of 70% methanol was added to the flask, which was then sealed and weighed. The solution was ultrasonically extracted (250 W, 40 kHz) for 50 min, cooled, reweighed, and brought to the original weight with 70% methanol. It was then shaken well and passed through a 0.22 μm membrane, thus obtaining the test solution, which was injected and measured at 0, 8, 16, 24, 32, 40, and 48 h. The relative standard deviation (RSD) of the peak areas of the eight reference substances ranged from 0.31% to 2.06%, indicating good stability of the test solution within 48 h. Precision weighing of known-content XXD freeze-dried powder was followed by the addition of liquiritin (0.21391 mg/mL), spinosin (0.0615 mg/mL), hesperidin (0.21391 mg/mL), ginsenoside Rg1 (1.697227 mg/mL), ginsenoside Re (0.746067 mg/mL), β-asarone (0.026484 mg/mL), ginsenoside Rb1 (0.621261 mg/mL), and ammonium glycyrrhizinate (0.138041 mg/mL). The solution was then injected and measured; and the average sample recovery rates were calculated as 96.69%, 100.14%, 102.73%, 102.97%, 100.06%, 103.15%, 96.33%, and 96.53% for liquiritin, spinosin, hesperidin, ginsenoside Rg1, ginsenoside Re, β-asarone, ginsenoside Rb1, and ammonium glycyrrhizinate, respectively, with RSDs of 0.26%–2.59%, indicating that this method showed good accuracy. The content of the eight metabolites in the samples was calculated (Table 3).

**TABLE 3:** Contents of eight main metabolites in XXD freeze-dried powder (mg/g).

Serial number	Liquiritin	Spinosin	Hesperidin	Ginsenoside Rg1	Ginsenoside Re	β-asarone	Ginsenoside Rb1	Ammonium glycyrrhizinate
S1	0.034091	0.046250694	0.182789	1.263342334	0.785716557	0.018897	0.419318	0.122108
S2	0.03758	0.052251149	0.201823	1.393430542	0.865617764	0.022877	0.491655	0.13529
S3	0.036532	0.060267395	0.229851	1.313783631	0.766150044	0.025839	0.523932	0.132669
S4	0.036783	0.061902926	0.231003	1.323181468	0.855789206	0.025532	0.560824	0.133167
S5	0.052649	0.080738224	0.23488	1.5223532	0.553456418	0.032631	0.468437	0.172148
S6	0.053599	0.079642728	0.267564	1.561168356	0.578036723	0.037313	0.477856	0.172504
S7	0.033923	0.059124637	0.421689	1.48674555	0.588972245	0.027454	0.546249	0.115218
S8	0.037423	0.066186777	0.463668	1.588738722	0.629773721	0.030212	0.594219	0.129175
S9	0.037509	0.065433511	0.46516	1.925405151	0.895773844	0.030199	0.566533	0.127523
S10	0.046577	0.066175291	0.429965	2.084475222	0.752984027	0.028797	0.384752	0.152622

### 2.4 Medicinal herbs and preparation

This study used concentrated botanical drug granules, and each 1 g of the concentrated botanical drug granules corresponded to the following botanical drug decoction pieces: 3.3 g of Panax ginseng, 10 g of Poria cocos, 10 g of Ziziphus jujuba, 12 g of Pinellia ternata, 12 g of Citrus reticulata, 10 g of Massa Medicata Fermentata, 3 g of Aconitum carmichaelii, 6 g of Acorus tatarinowii, and 3 g of Glycyrrhiza uralensis. The XXD granules were purchased from China Resources Sanjiu Medical and Pharmaceutical Co., Ltd., a manufacturer holding a Good Manufacturing Practice (GMP) certificate, ensuring the stability of the medication. The granules were produced with the following batch numbers: Panax ginseng Lot: 2110005C, Poria cocos Lot: 2109003C, Ziziphus jujuba Lot: 2170003C, Pinellia ternata Lot: 2109003C, Citrus reticulata Lot: 2109002S, Massa Medicata Fermentata Lot: 2104006S, Aconitum carmichaelii Lot: 2109001S, Acorus tatarinowii Lot: 2108002S, Glycyrrhiza uralensis Lot: 2110006C. Each packet contains 19.5 g of granules (equivalent to 132 g of botanical drug). The granules were mixed according to the dosage of the raw herbs, dissolved in 60°C distilled water, heated and stirred constantly, and prepared into a solution with a concentration of 88.725 mg/mL, and stored at 4°C in a sealed container.

Preparation of XXD-Medicated Serum: Following a 7-day acclimation period, 20 rats were randomly divided into two groups (n = 10 each): blank serum group and XXD-medicated serum group. The rat dosage, calculated using the Human-Animal Body Surface Area Conversion Table (Huang et al., 2004), was established at 3.375 g kg^-1^·d^-1^. Control group animals received an equivalent volume of saline solution. Oral gavage was administered daily for a week. Two hours post-final dose, the rats were anesthetized using intraperitoneal sodium pentobarbital. Blood was then collected aseptically from the abdominal aorta and allowed to coagulate for 2 h. After centrifugation, the serum was pooled by group, heat-inactivated (56°C, 30 min), sterile filtered, and cryopreserved at −80°C for subsequent use.

The probiotic freeze-dried powder, 3 g/bag, was purchased from Weikaihaien (Shandong) Biological Engineering Co., Ltd., Lot: 2022022. It was dissolved in distilled water at room temperature, shaken well, and prepared into a solution with a concentration of 0.0078 mg/mL, and stored at 4°C in a sealed container.

### 2.5 *In vitro* experimental protocol

#### 2.5.1 Coculture of hCMEC/D3 and HBVP cells

A Transwell coculture system was utilized to construct an *in vitro* BBB model. The hCMEC/D3 and HBVP cells were seeded into the upper and lower chambers, respectively, of a six-well Transwell plate at a 1:1 ratio and cultured until they reached more than 85% confluence. After digestion and counting, the hCMEC/D3 and HBVP cells were added to their respective chambers at a 1:1 volume ratio, and the culture medium was added to the appropriate level. After 48 h of coculture, a 4-h liquid surface leakage test was performed to evaluate the BBB model’s establishment. After changing the culture medium, if the liquid surface difference was greater than 0.5 cm, the culture continued for 4 h. If the liquid surface difference remained unchanged, the *in vitro* BBB model was considered to be preliminarily established.

#### 2.5.2 Lipopolysaccharide stimulation of hCMEC/D3 and HBVP cells

Lipopolysaccharide (LPS), a constituent of the outer membrane of Gram-negative bacteria, is commonly used to induce neuroinflammation (Yu et al., 2024). After successfully establishing the *in vitro* BBB model, LPS was used to induce BBB damage. Logarithmically growing hCMEC/D3 and HBVP cells were seeded into 96-well plates. After 8 h of incubation, 100 μL of a diluted LPS solution (initial concentration of 100 mg/mL) was added to each well at final concentrations of 0.5, 1, 1.5, and 2 μg/mL. Cell viability was evaluated at 24, 48, and 72 h to determine the optimal culture time and concentration.

#### 2.5.3 XXD-medicated serum intervention and grouping

The LPS-induced hCMEC/D3 and HBVP cells were treated with 5%, 10%, and 20% XXD-medicated serum, and cell viability was calculated to screen for the optimal concentration. The successfully established *in vitro* BBB models were subdivided into the following groups: control group, LPS group, 24-h XXD-medicated serum group, 48-h XXD-medicated serum group, 72-h XXD-medicated serum group, and blank serum group.

#### 2.5.4 Western blot detection of BBB transport-related protein expression

Logarithmically growing hCMEC/D3 and HBVP cells were modeled, grouped, administered, and incubated as described above. Following this, Western blotting experiments were performed. The cells were collected, centrifuged, lysed, and centrifuged again to collect the supernatant. Protein levels were determined via the BCA assay. Each well was loaded with 20 μg of total protein. Proteins were separated by SDS-PAGE (6% stacking gel at 60 V, 10% separating gel at 90 V), followed by wet transfer at 300 mA for 90 min. Subsequently, the samples were blocked using 5% skim milk on a shaker for 1 h and subsequently incubated with primary antibodies (antibodies against P-glycoprotein [P-gp], cannabinoid receptor types 1 and 2 [CB1 and CB2], and major facilitator superfamily domain-containing protein 2a [Mfsd2a]) at a 1:5000 dilution at 4°C overnight with shaking. Following three washes in TBST, secondary antibodies were applied at room temperature for 90 min. Subsequently, TBST washing was performed three times, ECL reagent was used for darkroom exposure, and band gray values were analyzed using ImageJ software (v 1.8.0).

#### 2.5.5 RT-PCR detection of P-gp mRNA

The *in vitro* BBB model was incubated, then removed, digested, and centrifuged. RNA extraction was performed with TRIzol, followed by cDNA synthesis using a reverse transcription kit. RT-PCR was conducted according to the manufacturer’s instructions. The amplified products were separated via 1% agarose gel electrophoresis and imaged using an image analyzer. Expression levels of mRNA were determined using the 2^−ΔΔCT^ method. Primer details are provided in Table 4.

**TABLE 4:** Rt-PCR primer sequences.

Gene	Primer sequence (5′∼3′)	Product length (bp)
P-gp	Forward: GAC​ATG​ACC​AGG​TAT​GCC​TAT​TAT​T	160
	Reverse: ACC​AGC​CTA​TCT​CCT​GTC​GCA​T	
GAPDH	Forward: GGA​AGC​TTG​TCA​TCA​ATG​GAA​ATC	168
	Reverse: TGA​TGA​CCC​TTT​TGG​CTC​CC	

#### 2.5.6 Immunofluorescence detection of P-gp expression

The incubated *in vitro* BBB model was extracted post-incubation, PBS-washed, and fixed with 4% paraformaldehyde. Membrane permeabilization was achieved using Triton X-100, followed by BSA blocking (37°C, 1 h). Sequential antibody incubations were performed: primary (overnight) and secondary (1 h). Nuclear counterstaining employed DAPI, and the samples were subsequently imaged using a fluorescence microscope at a magnification of ×20.

### 2.6 *In vivo* experimental protocol

#### 2.6.1 Animal grouping and drug administration

SAMR1 mice were designated as the control group (Control), while SAMP8 mice were randomly divided into the model group (Model), the probiotics group (Probiotics), the high-dose Xixin Decoction granules group (H-XXD), the medium-dose Xixin Decoction granules group (M-XXD), and the low-dose Xixin Decoction granules group (L-XXD), with eight mice in each group. The dosages were calculated based on the equivalent clinical dosages and the body surface area of the animals (Huang et al., 2004). The probiotics group received a dose of 0.39 g·kg^-1^·d^-1^, the M-XXD group received a dose of 2.535 g·kg^-1^·d^-1^, the H-XXD group received twice the equivalent clinical dose at 5.07 g·kg^-1^·d^-1^, and the L-XXD group received half the equivalent clinical dose at 1.2675 g·kg^-1^·d^-1^. The treatment was administered continuously for 8 weeks. The drugs were dissolved in distilled water to prepare solutions of the appropriate concentrations. Each treatment group received the drugs by gavage daily at the designated dose, with a volume of 10 mL·kg^-1^·d^-1^. Equivalent volumes of distilled water were administered to both the control and model groups via gavage.

#### 2.6.2 Behavioral testing

The Morris water maze test was performed for six consecutive days after administration. The initial 5 days consisted of place navigation trials. Mice were released into the water pool from four different quadrants each day, facing the wall. The escape latency, defined as the time required to find the submerged platform, was recorded with a limit of 60 s per trial. Upon reaching the platform, mice were permitted a 10-s rest period. If mice were unable to find the platform within the allotted time, they were gently guided to it, allowed a 10-s rest, and assigned a maximum escape latency of 60 s. On the sixth day, a spatial probe test was conducted by removing the hidden platform and releasing mice from the first quadrant, facing the pool wall. They were allowed to swim freely for 60 s. During this trial, the time spent in the target quadrant and frequency of crossing the former platform site were recorded.

#### 2.6.3 BBB permeability testing

Following the final behavioral test, four mice per group were randomly chosen and injected with a 2% Evans blue solution (4 mL/kg) through the tail vein. Specific methods referred to the published literature (Schoch et al., 2014). Upon turning blue in limbs and eyes, the mice were anesthetized via intraperitoneal injection of 0.5% sodium pentobarbital (0.1 mL/g). The specific methods were performed with reference to the literature (Levin-Arama et al., 2016). Cardiac perfusion was performed through the left ventricle with physiological saline until the liver and kidneys turned white and clear fluid flowed from the right atrium. This was followed by rapid perfusion with 4% paraformaldehyde precooled to 4 °C, which was maintained at a slow and steady rate for 10–15 min. The brain tissue was subsequently collected, fixed in 4% paraformaldehyde (4°C, 12 h), embedded in OCT, rapidly frozen at −20°C, and sectioned coronally at 20 μm. Evans blue extravasation was observed under a laser confocal microscope and quantified using ImageJ 1.8.0 software.

#### 2.6.4 ELISA detection of hippocampal Aβ_1-42_ content and levels of neuroinflammatory factors

Hippocampal tissue was precooled on ice and homogenized in nine times the volume of the PBS solution, and the supernatant was collected. Following the instructions provided with the ELISA kit, the levels of Aβ_1-42_, NF-κB p65, NLRP3, TNF-α, and IL-1β were measured. Absorbance at 450 nm was assessed using an enzyme-labeled instrument.

#### 2.6.5 Immunofluorescence detection of BBB transport-related protein receptors and microglial activation markers in the hippocampal CA1 region

Anesthesia was administered to the mice via intraperitoneal injection of 0.5% sodium pentobarbital. A thoracic incision exposed the heart, allowing for left ventricular needle insertion and right atrial appendage excision. Perfusion commenced with physiological saline until clear fluid emerged from the oronasal cavities, followed by 4% paraformaldehyde perfusion until complete body rigidity. The intact brain was extracted and underwent a series of preparatory steps: 24-h fixation in 4% paraformaldehyde, ethanol dehydration, xylene clearing, and paraffin embedding. Five-micrometer sections were prepared and subsequently washed thrice with PBS before blocking with 3% bovine serum albumin (37°C, 30 min). Primary antibody incubation (targeting P-gp, CB1, CB2, RAGE, IBA1, TREM2, TLR1, and TLR2) was conducted overnight at 4°C. Following PBS washing, sections were incubated with fluorescent secondary antibodies (37°C, 1 h). After additional PBS washes, DAPI staining was performed, and sections were mounted using an anti-fluorescence quenching medium. Imaging was performed using confocal microscopy at a magnification of ×40, and relative fluorescence intensity was quantified with ImageJ software (version 1.8.0).

#### 2.6.6 Western blot detection of BBB transport-related protein receptors and neuroinflammatory factors in mouse hippocampal tissue

Hippocampal tissue was extracted, lysed, and homogenized ultrasonically on ice. Post-lysis centrifugation at 12,000 × g for 15 min at 4°C, the supernatant was collected for BCA protein quantification. Each well was loaded with 20 μg of total protein. Proteins underwent SDS-PAGE separation (6% stacking gel, 60V; 10% separating gel, 90V) and wet transfer (300mA, 90min). Membranes were blocked with 5% skim milk (1h, shaking) and incubated overnight with primary antibodies (anti-P-gp, CB1, CB2, RAGE, IBA1, TLR1, TLR2, TREM2, Mfsd2a, MRP2, LRP1, and CMPK2) at 4°C under agitation. After TBST washing, secondary antibody incubation was performed (90min, room temperature). ECL-based chemiluminescence detection was conducted in darkness. Band intensities were analyzed using ImageJ (v1.8.0).

### 2.7 Data analysis

Statistical analyses were conducted using GraphPad Prism 9.0 software. Data are presented as mean ± SD. Levene’s test assessed variance homogeneity. One-way ANOVA was applied for multiple group comparisons, followed by LSD-t tests for data with significant differences. A *p*-value <0.05 was considered statistically significant.

## 3 Results

### 3.1 *In vitro* experimental results

#### 3.1.1 Optimal concentration screening for LPS and XXD-medicated serum

**FIGURE 2:**
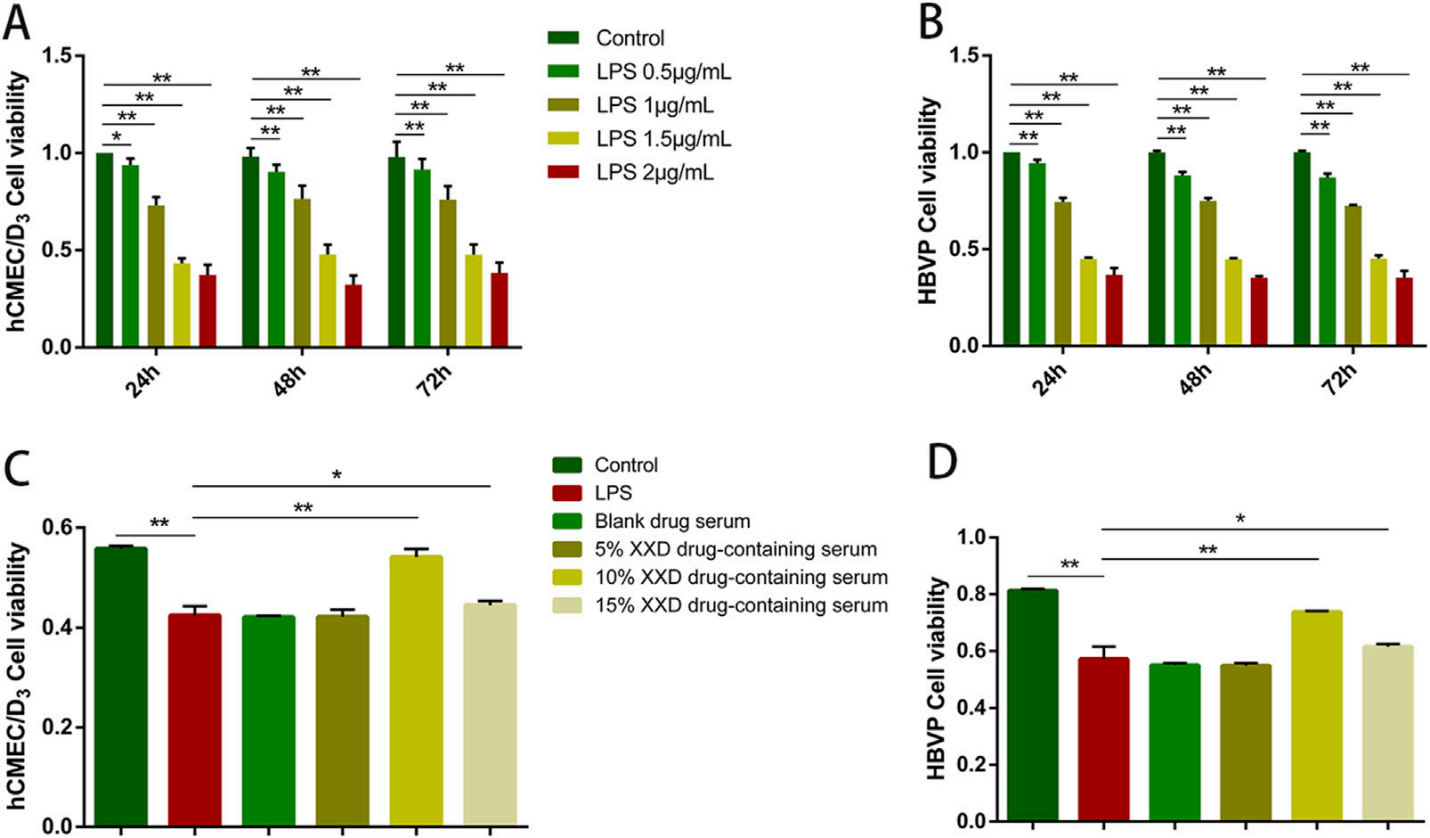
Screening of optimal concentrations of LPS and XXD medicated serum. **(A)** Effects of different concentrations of LPS on the viability of hCMEC/D3 cells after 24, 48, and 72 h of incubation; **(B)** Effects of different concentrations of LPS on the viability of HBVP cells after 24, 48, and 72 h of incubation; **(C)** Effects of different concentrations of XXD medicated serum on the viability of LPS-induced hCMEC/D3 cells; **(D)** Effects of different concentrations of XXD medicated serum on the viability of LPS-induced HBVP cells. MTT assay; **p < 0.01; *p < 0.05.

LPS can disrupt the BBB through various pathways, and damage to the BBB can contribute to the progression of a range of diseases, with AD being a notable example (Peng et al., 2021). Therefore, this study utilized LPS to establish a BBB inflammation model. Optimal LPS concentration was determined through cell viability assessment using the MTT assay. The results showed that, after treating hCMEC/D3 and HBVP cells with 0.5–2 μg/mL LPS, cell viability exhibited a notable decline in comparison to the control group and exhibited a negative correlation with the LPS concentration. As high concentrations of LPS could cause irreversible cell damage, 1 μg/mL LPS was selected for subsequent experiments. On comparing the cell viability of hCMEC/D3 and HBVP cells exposed to 1 μg/mL LPS for 24, 48, and 72 h, it was found that cell viability in the 48-h and 72-h groups exhibited no statistically significant difference from that in the 24-h group (Figures 2A, B). Therefore, a 24-h treatment with 1 μg/mL LPS was used as the modeling condition.

After the administration of 1 μg/mL LPS to hCMEC/D3 and HBVP cells for 24 h, different concentrations of XXD-medicated serum were added for co-incubation. The MTT assay results indicated that 1 μg/mL LPS significantly inhibited cell proliferation, while 10% XXD-medicated serum significantly reversed this effect and increased cell viability (Figures 2C, D). Additionally, the 15% concentration of XXD-medicated serum did not show any further improvement in cell viability. This observation suggests that the 15% concentration may have reached a saturation point of the pharmacological effect or potentially introduced additional adverse effects, thereby failing to further enhance cell viability. Therefore, 10% XXD-medicated serum was chosen for subsequent experiments, as it exhibited the best efficacy in restoring cell viability while avoiding the potential drawbacks associated with higher concentrations.

#### 3.1.2 XXD-medicated serum promotes P-gp expression in the *in vitro* BBB model

The BBB can be described as a highly evolved CNS microvascular functional unit, comprising CNS endothelial cells, neural progenitor cells, pericytes, astrocytes, and other neural and immune cells, in addition to various functional protein structures (Obermeier et al., 2013). These components form the neurovascular unit (NVU) that maintains BBB function (Huang et al., 2020). Pericytes envelop the abluminal surface of cerebral vascular walls, including capillaries, precapillary arterioles, and postcapillary venules. The highest pericyte coverage rate is seen in the neural tissue among the capillary beds of different organs (Allt and Lawrenson, 2001). Pericytes are crucial in promoting vascular stability and maintaining BBB integrity (Winkler et al., 2011). Therefore, this study employed hCMEC/D3 and HBVP cells to construct an *in vitro* BBB model.

**FIGURE 3:**
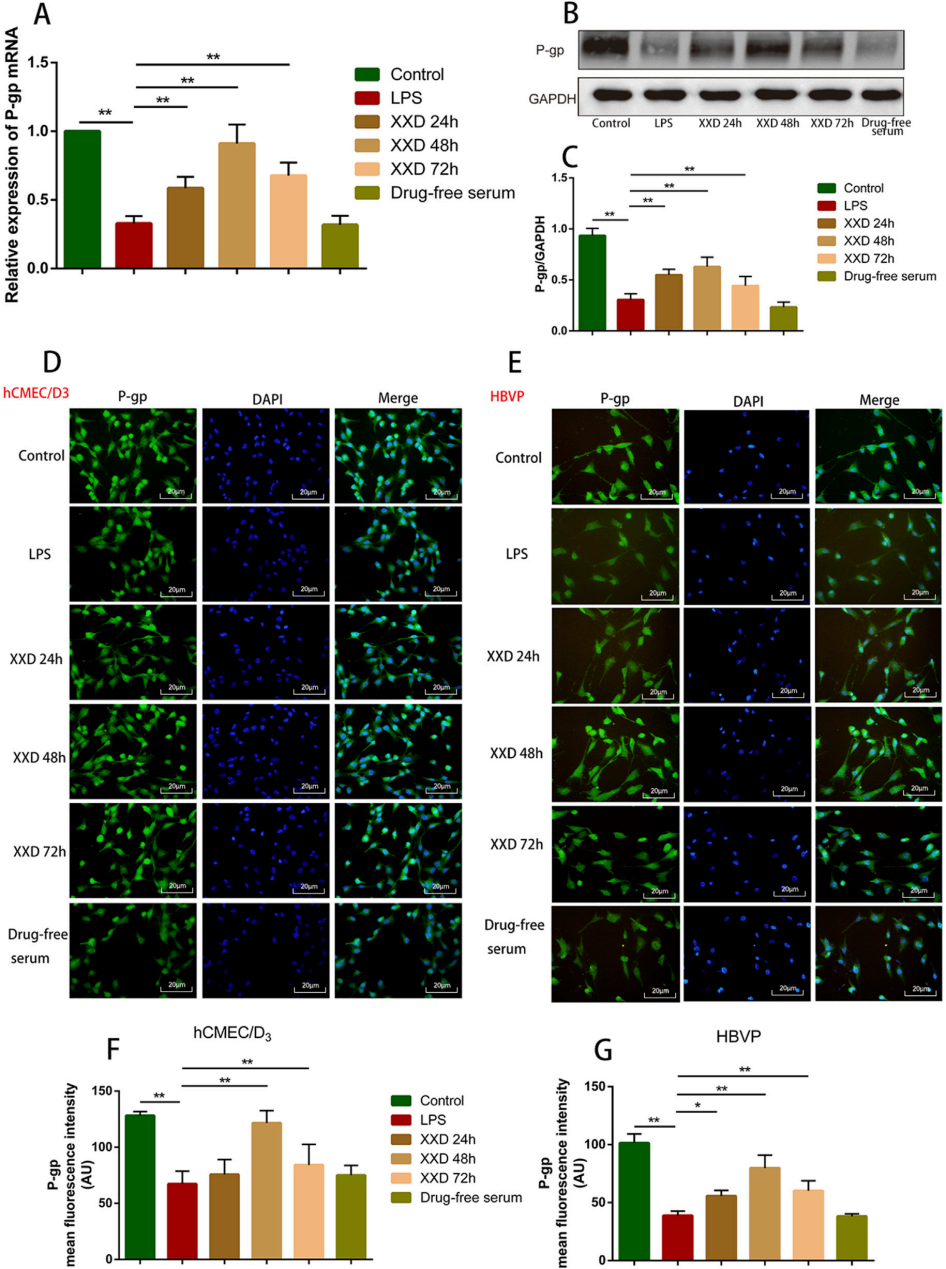
XXD medicated serum promotes P-gp expression in the blood-brain barrier cell model. **(A)** Comparison of P-gp mRNA expression; **(B)** and **(C)** Comparison of relative P-gp protein expression; **(D)** and **(F)** Comparison of P-gp protein positive expression in hCMEC/D3 cells; **(E)** and **(G)** Comparison of P-gp protein positive expression in HBVP cells. Scale bar: 20 μm; images at ×200 magnification; **p < 0.01, *p < 0.05.

P-gp, an ABC transporter encoded by the human MDR1 gene, serves as a crucial efflux mechanism in the BBB. It inhibits substrate drug entry into the brain while promoting the elimination of endogenous molecules, including Aβ—a hallmark pathological feature of AD (Pyun et al., 2022). This study revealed that, relative to the control group, LPS treatment significantly downregulated both mRNA and protein expression of P-gp, indicating that P-gp expression is reduced when the BBB is damaged. Relative to the LPS group, the XXD-medicated serum upregulated P-gp mRNA and protein expression after 24, 48, and 72 h of culture, with the most significant effect observed at 48 h (Figure 3), suggesting that XXD can improve BBB transport dysfunction by upregulating P-gp expression.

#### 3.1.3 XXD-medicated serum upregulates CB2 and Mfsd2a expression and downregulates CB1 expression in the in vitro BBB model

Endocannabinoids, originating from dietary omega-3 and omega-6 polyunsaturated fatty acids, are endogenous bioactive lipids. The endocannabinoid system (ECS) is characterized by two primary receptors: CB1 and CB2. CB1 is predominantly expressed in CNS presynaptic regions (Zou and Kumar, 2018). It modulates cytokine release both within and outside the CNS and immune cell migration, with expression levels influenced by cell activation and stimulus type (Paul et al., 2012). CB2 is predominantly expressed in peripheral immune cells and brain tissue (Nagarkatti et al., 2009). Primarily serving an immunomodulatory function, it regulates immune cell migration and cytokine release both within and beyond the immune system (Massi et al., 2006). During neuroinflammation or BBB dysfunction, P-gp regulates ECS-related receptor protein expression, thereby influencing the inflammatory process. Regulating the P-gp/ECS axis can alleviate BBB dysfunction and neuroinflammation. Mfsd2a, a member of the major facilitator superfamily (MFS), plays a critical role in BBB integrity and DHA transport. Mfsd2a-deficient mice exhibit significantly lower brain DHA levels, resulting in neuronal loss and cognitive impairment due to DHA’s crucial role in brain development and maintenance. Mfsd2a inhibits CNS endothelial cell transport and mitigates BBB damage. Consequently, it has gained prominence in neurological disease research (Huang and Li, 2021).

**FIGURE 4:**
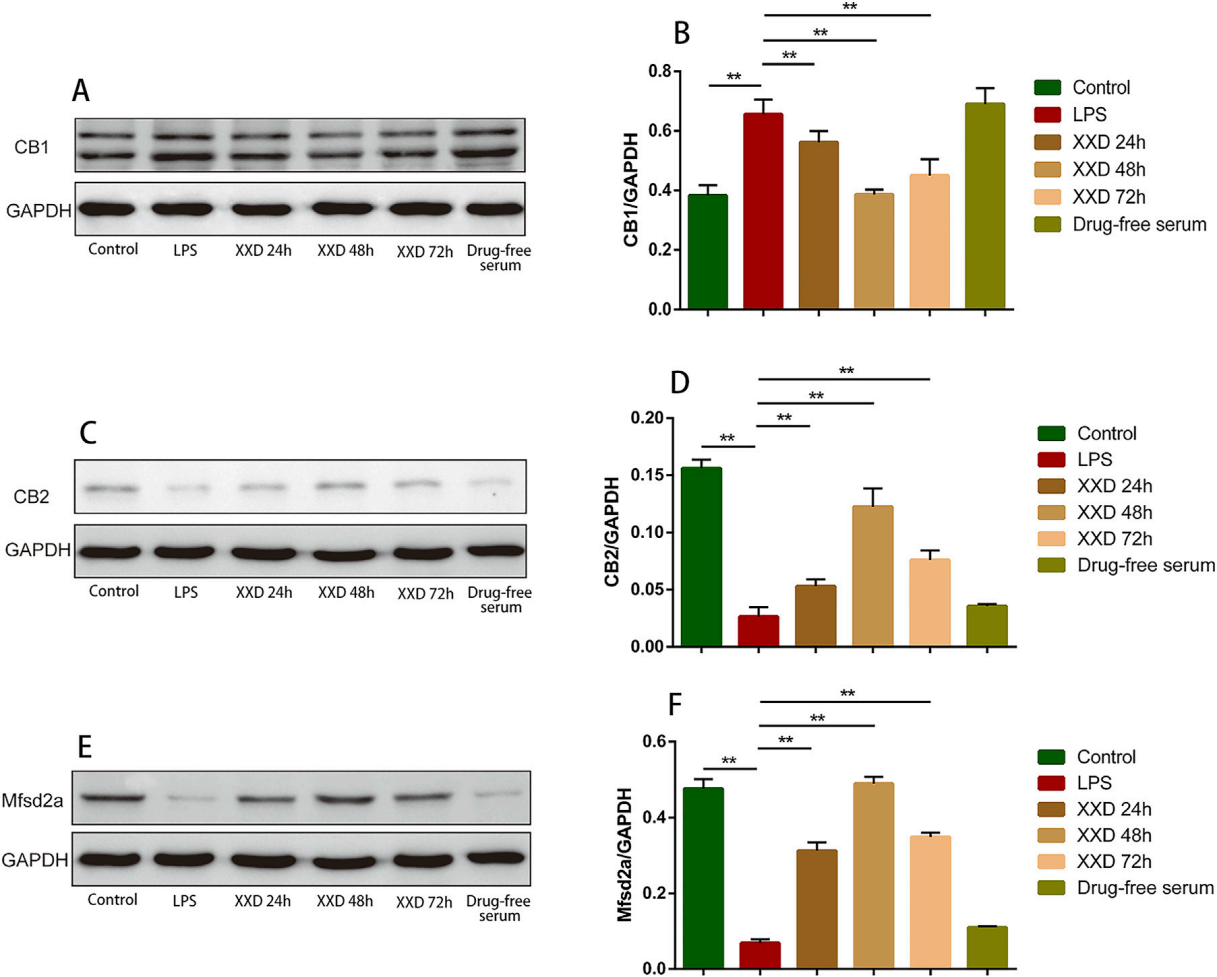
XXD medicated serum upregulates CB2 and Mfsd2a expression and downregulates CB1 expression in the blood-brain barrier cell model. **(A)** and **(B)** Comparison of relative CB1 protein expression; **(C)** and **(D)** Comparison of relative CB2 protein expression; **(E)** and **(F)** Comparison of relative Mfsd2a protein expression; **p < 0.01, *p < 0.05.

This study demonstrated that, relative to the control group, LPS treatment upregulated CB1 protein expression while downregulating both CB2 and Mfsd2a expression. indicating that neuroinflammation can lead to an abnormal ECS as well as abnormal Mfsd2a expression, resulting in BBB dysfunction. Compared with the LPS group, XXD-medicated serum downregulated CB1 expression and upregulated CB2 and Mfsd2a expression after incubation for 24, 48, and 72 h (Figure 4), suggesting that XXD can improve BBB dysfunction by modulating the expression of the transport-related protein Mfsd2a and the P-gp/ECS axis.

### 3.2 *In vivo* experimental results

#### 3.2.1 XXD improves learning and spatial memory abilities and reduces evans blue extravasation in SAMP8 mice

SAMP8 mice serve as an optimal AD model, demonstrating age-associated learning and memory impairments, in addition to exhibiting the majority of pathological characteristics associated with AD pathogenesis, including oxidative stress, inflammation, and Aβ deposition. Therefore, SAMP8 mice aid in visualizing the effects of AD and provide an effective method for finding new therapeutic targets (Liu et al., 2020).

**FIGURE 5:**
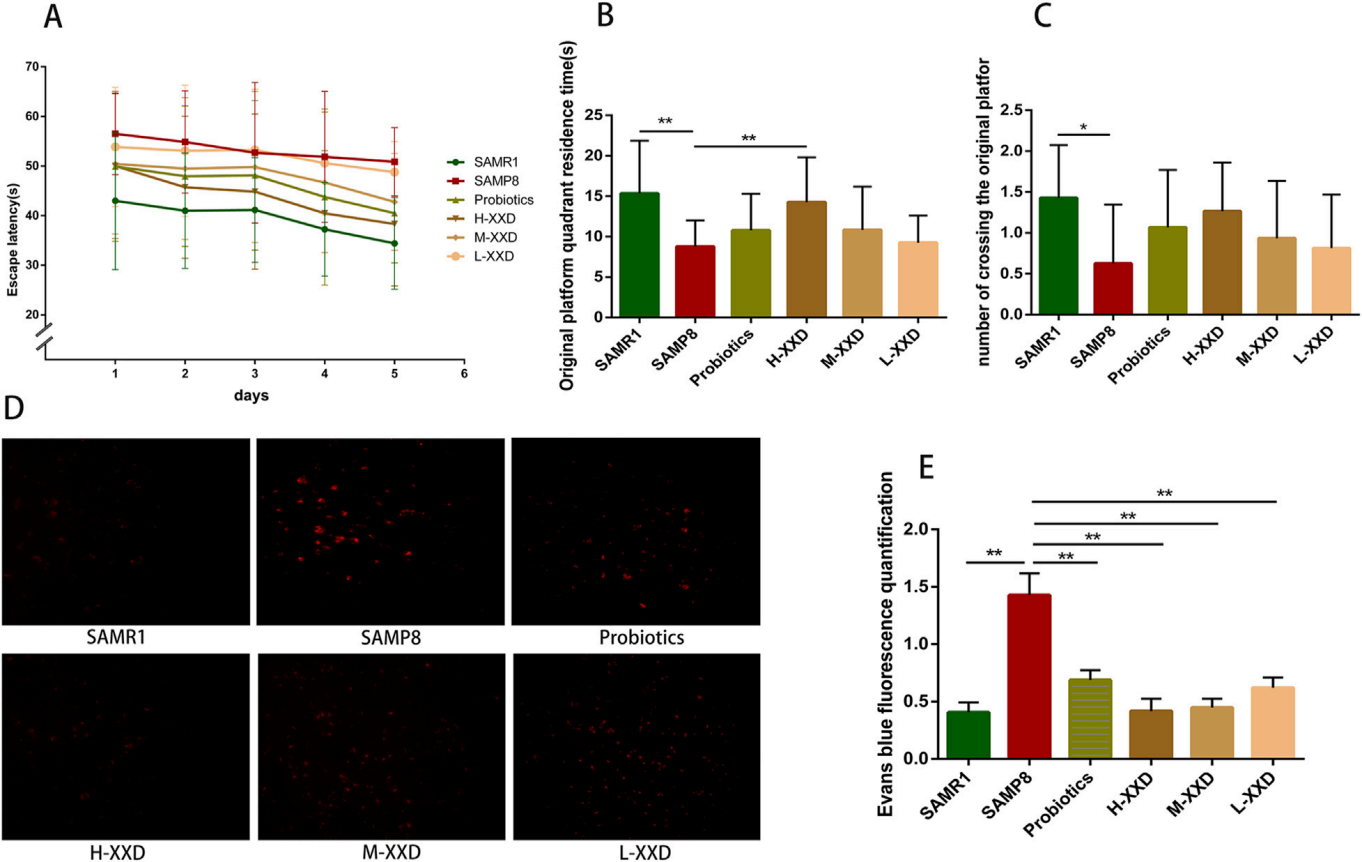
XXD improves learning and spatial memory impairments in SAMP8 mice and reduces Evans blue extravasation. **(A)** Escape latency (s) in the place navigation test (n = 8); **(B)** Time spent in the target quadrant (s) in the spatial probe test (n = 8); **(C)** Number of crossings over the platform location in the spatial probe test; **(D)** and **(E)** Comparison of Evans blue extravasation in the brains of different groups of mice (n = 4); images at ×400 magnification; **p < 0.01, *p < 0.05.

The Morris water maze is a commonly used behavioral test for assessing spatial cognition in rodents (Vorhees and Williams, 2006). This study investigates, compared to SAMR1 controls, SAMP8 mice exhibited significantly prolonged escape latencies over five consecutive days, along with decreased target quadrant dwell time and crossings, indicating marked spatial cognitive impairment. High-dose XXD treatment, relative to untreated SAMP8 mice, significantly reduced escape latencies throughout the 5-day period and increased target quadrant residence time. Medium- and low-dose XXD groups also demonstrated significantly shortened escape latencies (Figures 5A–C). These results suggest XXD’s potential to ameliorate spatial cognitive deficits in SAMP8 mice.

Evans blue, a widely utilized azo dye, is often used for tracing and observing BBB integrity. Under normal conditions, Evans blue, which is bound to plasma albumin, is unable to pass through the BBB and thus is incapable of staining the nervous system. However, if the BBB is disrupted, Evans blue is able to penetrate and cause staining (Liu et al., 2018). In this study, Evans blue extravasation was markedly increased in the brain tissue of SAMP8 mice, indicating BBB damage and increased permeability. Compared with the SAMP8 group, Evans blue extravasation in the brain tissue of XXD-treated groups significantly decreased (Figures 5D, E). Therefore, XXD may reduce BBB permeability by improving its ultrastructure, although the specific mechanisms require further investigation.

#### 3.2.2 XXD regulates BBB transport-related proteins and promotes Aβ_1-42_ clearance in SAMP8 mice

##### 3.2.2.1 XXD regulates the P-gp/ECS axis

**FIGURE 6:**
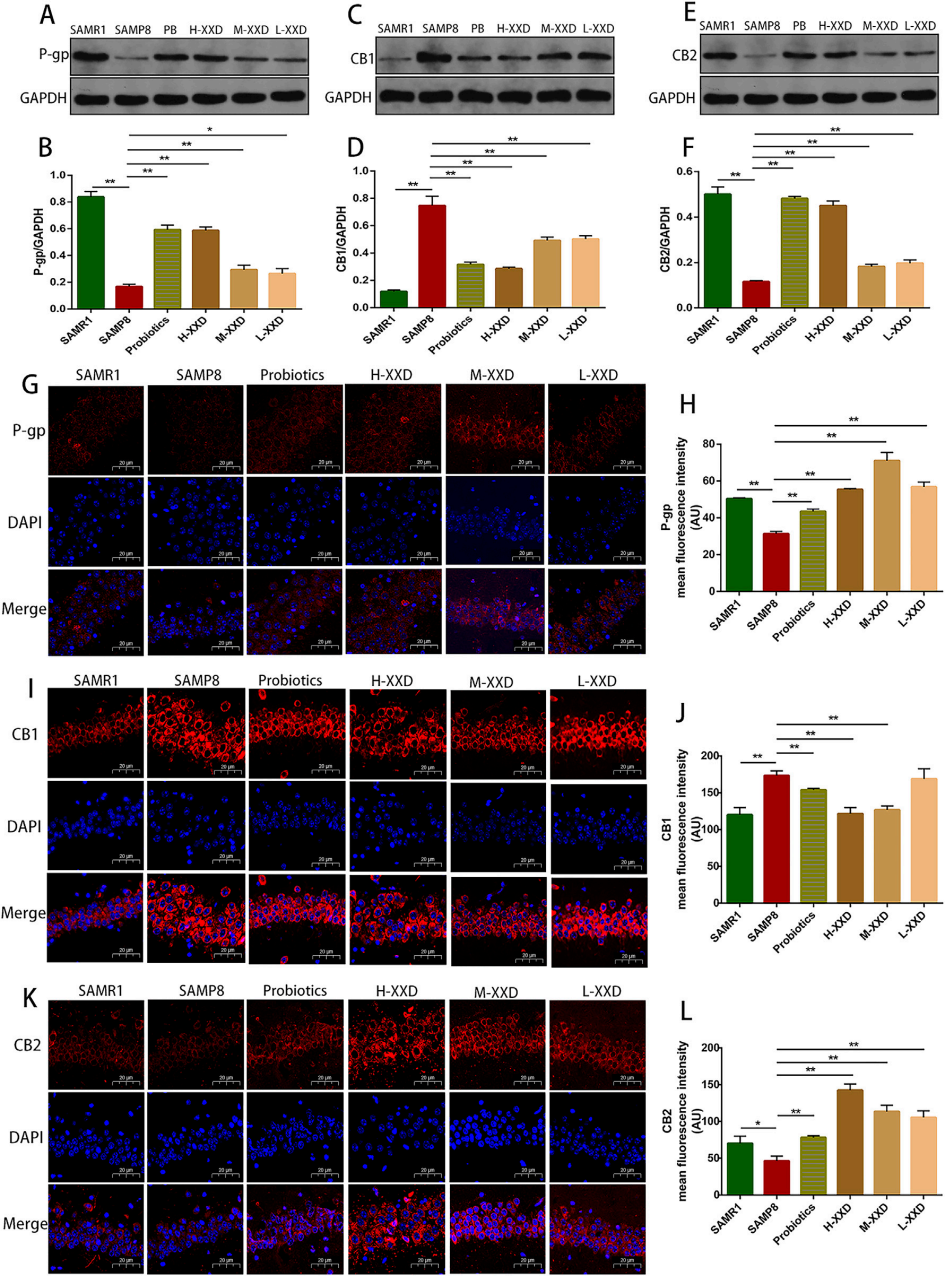
XXD Modulates the P-gp/eCBs Axis in the Hippocampal CA1 Region of SAMP8 Mice; **(A)** and **(B)** Comparison of relative P-gp protein expression levels; **(C)** and **(D)** Comparison of relative CB1 protein expression levels; **(E)** and **(F)** Comparison of relative CB2 protein expression levels; **(G)** and **(H)** Immunohistochemical staining for P-gp in the hippocampal CA1 region of mice; **(I)** and **(J)** Immunohistochemical staining for CB1 in the hippocampal CA1 region of mice; **(K)** and **(L)** Immunohistochemical staining for CB2 in the hippocampal CA1 region of mice; (n = 6); scale bar: 20μm; images captured at ×400 magnification; **p < 0.01, *p < 0.05.

**FIGURE 7:**
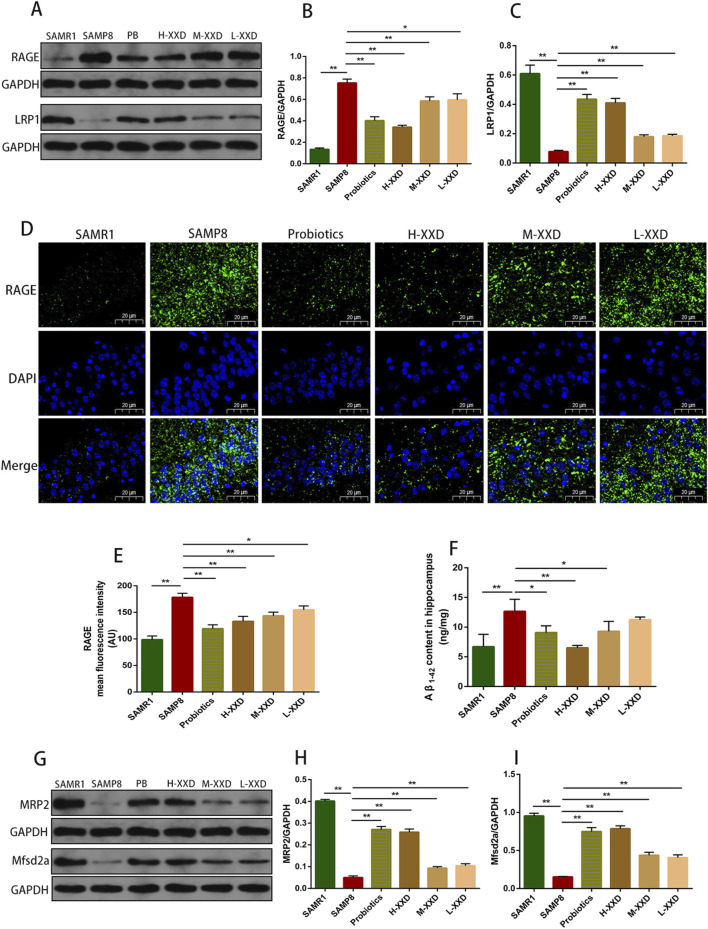
XXD Regulates the RAGE/LRP1 Receptor System and MRP2, Mfsd2a Proteins, Accelerating Aβ_1-42_ Clearance in the Brains of SAMP8 Mice; **(A)**, **(B)**, and **(C)** Comparison of relative RAGE and LRP1 protein expression levels; **(D)** and **(E)** Comparison of RAGE protein positive expression in the hippocampal CA1 region; **(F)** Comparison of hippocampal Aβ_1-42_ content; **(G)**, **(H)**, and **(I)** Comparison of relative MRP2 and Mfsd2a protein expression levels; (n = 6); Scale bar: 20μm; images captured at ×400 magnification; **p < 0.01, *p < 0.05.

In a healthy CNS, Aβ production and clearance rates have been observed to be 7.6% and 8.3% per hour (Bateman et al., 2006). P-gp plays a pivotal role in the normal clearance of Aβ through the BBB, with its expression and function negatively related to aging, Aβ deposition, and AD. Hartz and colleagues demonstrated that inhibiting P-gp reduces Aβ_42_ transport across brain capillary lumens (Hartz et al., 2010). The present research demonstrated that P-gp fluorescence intensity and protein expression in the hippocampal CA1 region of SAMP8 mice significantly decreased, while Aβ_1-42_ content significantly increased, indicating a negative correlation between hippocampal Aβ accumulation and P-gp expression in SAMP8 mice. In comparison to the SAMP8 group, the P-gp fluorescence intensity and protein expression in the hippocampal CA1 region of XXD-treated mice in all dosage groups significantly increased (Figure 6A, B, G, H), while hippocampal Aβ_1-42_ content significantly decreased (Figure 7F), indicating that XXD can upregulate P-gp expression and reduce Aβ accumulation.

The ECS is a widespread neuroregulatory network that undergoes changes and functional decline in many neurological diseases, potentially serving as a key component in regulating neuroinflammation and the pathogenesis of neurodegenerative diseases (Stella, 2010). The ECS comprises endocannabinoids, cannabinoid receptors CB1 and CB2, and proteins participating in endocannabinoid metabolism. The primary endogenous agonists of CB1 and CB2 are 2-arachidonoylglycerol (2-AG) and N-arachidonoylethanolamine (AEA) (Hillard, 2018). In the hippocampus of patients with AD, 2-AG levels are elevated, which has also been confirmed in rodent models (van der Stelt et al., 2006). Elevated endocannabinoids may boost the neuroprotective effects of CB1 and, via CB2 activation, inhibit microglial inflammation while stimulating Aβ phagocytosis (Papa et al., 2022). P-gp and ECS are closely related. Cannabidiol (CBD) can act through typical endocannabinoid receptors (CB1 and CB2) or various non-typical pathways, exerting an effect that inhibits the P-gp efflux function (Gómez et al., 2021). In intestinal inflammation models, P-gp knockdown or inhibition reduces the secretion of endocannabinoids, and the loss of CB2 exacerbates acute intestinal inflammation (Szabady et al., 2018). Therefore, it can be hypothesized that, in the CNS, P-gp may regulate ECS receptor expression, contributing to neuroinflammation or BBB dysfunction. In this study, the fluorescence intensity and expression of CB1 in the hippocampal CA1 region of SAMP8 mice increased, while the fluorescence intensity and expression of CB2 decreased, indicating a decline in ECS function in AD. Compared with the SAMP8 group, in all XXD-dose groups, the fluorescence intensity and expression of CB1 in the mouse hippocampal CA1 region decreased (Figures 6C, D, I, J), while the fluorescence intensity and expression of CB2 increased (Figures 6E, F, K, L), indicating that XXD has a regulatory effect on the ECS. Therefore, XXD may promote Aβ transport across the BBB and reduce Aβ accumulation in the brain by regulating the P-gp/ECS axis.

##### 3.2.2.2 XXD regulates the RAGE/LRP1 receptor system and MRP2 and Mfsd2a proteins

The transportation of Aβ across the BBB is mainly achieved through the RAGE/LRP1 receptor system. RAGE facilitates Aβ entry into the brain, while LRP1 mediates the removal of Aβfrom the brain. Blocking RAGE can decrease Aβ entry into the brain, inhibit NF-κB signaling and neuronal apoptosis, and improve cognitive function (Wang et al., 2018). LRP1 deficiency leads to reduced plasma Aβ, increased brain Aβ, and spatial memory deficits (Storck et al., 2016). In this study, in the hippocampal CA1 region of SAMP8 mice, the fluorescence intensity and protein expression of RAGE increased, LRP1 expression decreased, and Aβ content increased, suggesting that Aβ accumulation in the AD brain may have a relationship with the dysregulation of the RAGE/LRP1 receptor system in the BBB. Compared with the SAMP8 group, in all XXD-dose groups, RAGE expression decreased (Figure 7A, B, D, E), LRP1 expression increased (Figures 7A, C), and Aβ content decreased in the mouse hippocampal CA1 region (Figure 7F), suggesting that XXD potentially modulates RAGE/LRP1 receptor activity, thereby impeding Aβ influx into the brain while facilitating its efflux and elimination.

The ATP-binding cassette subfamily C member 1 (ABCC1), also known as multidrug resistance-associated protein 1 (MRP1), is a complete transporter exhibiting efflux capabilities. MRP1’s export function serves as a crucial pathway for Aβ elimination from cerebral tissue via the blood-brain barrier (BBB). Research has demonstrated that a lack of MRP1 results in elevated Aβ concentrations within the murine brain (Aykac and Sehirli, 2021). Similarly, MRP2/ABCC2 serves as an ATP-dependent efflux transporter present in the BBB (Girardin, 2006), potentially contributing to the clearance of Aβ from the brain. In this study, MRP2 expression in the hippocampal region of SAMP8 mice decreased, while Aβ content increased, suggesting that Aβ accumulation in the AD brain may have a correlation with MRP2 deficiency. Mice in the XXD treatment groups exhibited increased MRP2 expression (Figures 7G, H) and decreased Aβ content (Figure 7F), indicating that XXD may promote Aβ efflux by enhancing MRP2 activity.

Mfsd2a is a sodium-dependent lysophosphatidylcholine (LPC) symporter present in BBB endothelial cells (Huang and Li, 2021). Studies have shown that Mfsd2a-knockout mice exhibit increased endocytosis, leading to BBB leakage (Chow and Gu, 2017). Research on rats with chronic cerebral hypoperfusion has demonstrated that Mfsd2a overexpression can reduce BBB damage and cognitive decline (Shang et al., 2019). In this study, Mfsd2a protein expression in the hippocampal region of SAMP8 mice was reduced, whereas BBB permeability increased, indicating that BBB leakage in the AD brain may be linked to the reduced expression of the nutrient transporter Mfsd2a. Compared with the SAMP8 group, in all XXD-dose groups, Mfsd2a expression in the mouse hippocampal region increased (Figures 7G, I)and BBB permeability decreased, indicating that XXD may alleviate BBB damage by increasing Mfsd2a expression.

#### 3.2.3 XXD inhibits microglial activation and alleviates neuroinflammation in SAMP8 mice

As the primary CNS immune cells, microglia are pivotal in Alzheimer’s disease (AD). Upon activation, they can adopt either the neurotoxic pro-inflammatory M1 phenotype or the neuroprotective M2 state (Xie et al., 2021). Studies have shown that Aβ stimulation can upregulate the activity marker CD68 and M1 markers TNF-α, IL-1β, and CD86 in primary microglia (Shi et al., 2017). Injection of Aβ into the mouse hippocampus also increases levels of M1 microglia (Xie et al., 2020). IBA1 is a specific calcium-binding protein participating in microglial remodeling, regulating their migration, membrane ruffling, and phagocytosis, and is commonly used to assess microglial activation (Hendrickx et al., 2017).

TREM2 is specifically expressed in microglia and has anti-inflammatory effects (Qin et al., 2021). Suppression of TREM2-mediated signaling in microglial cells enhances the expression of TNF-α and nitric oxide synthase 2 (NOS2) genes. Conversely, elevated TREM2 levels lead to decreased transcription of TNF-α, IL-1β, and NOS2 (Takahashi et al., 2005). TLRs are crucial components of the neuroimmune system, exerting a pivotal influence on microglial activation and polarization. Activation of TLRs leads to NF-κB upregulation, promoting the synthesis of inflammatory mediators including cytokines (TNF-α, IL-1β, IL-6) and enzymes (iNOS, COX2). TLR2 forms a heterodimer with TLR1, enhancing pro-inflammatory cytokine production via the MyD88-dependent pathway and NF-κB activation. Studies show that increased TREM2 expression decreases specific TLRs (2, 4, 6) and pro-inflammatory mediators (IL-1β, TNF-α, IL-6) in microglia, thereby reducing Aβ_1-42_-induced neuroinflammatory responses (Long et al., 2019).

NLRP3 is a core component of the NLRP3 inflammasome, widely distributed in the CNS and highly expressed in microglia. NLRP3 recognizes exogenous or endogenous PAMPs and DAMPs, activates the downstream transcription factor NFκB, phosphorylates and degrades IκBα, facilitates NF-κBp65 nuclear translocation, initiating target gene transcription and upregulating NLRP3, IL-1β, and IL-18 precursor expression. Under PAMP and DAMP stimulation, the NLRP3 inflammasome assembles, releasing mature IL-1β and IL-18 and activating Gasdermin D, which causes microglial membrane rupture and the further release of more inflammatory factors (Hanslik and Ulland, 2020). CMPK2 is a newly discovered upstream factor of the NLRP3 inflammasome. It has been demonstrated that CMPK2 knockdown inhibits NLRP3 inflammasome activation (Chen et al., 2022).

**FIGURE 8:**
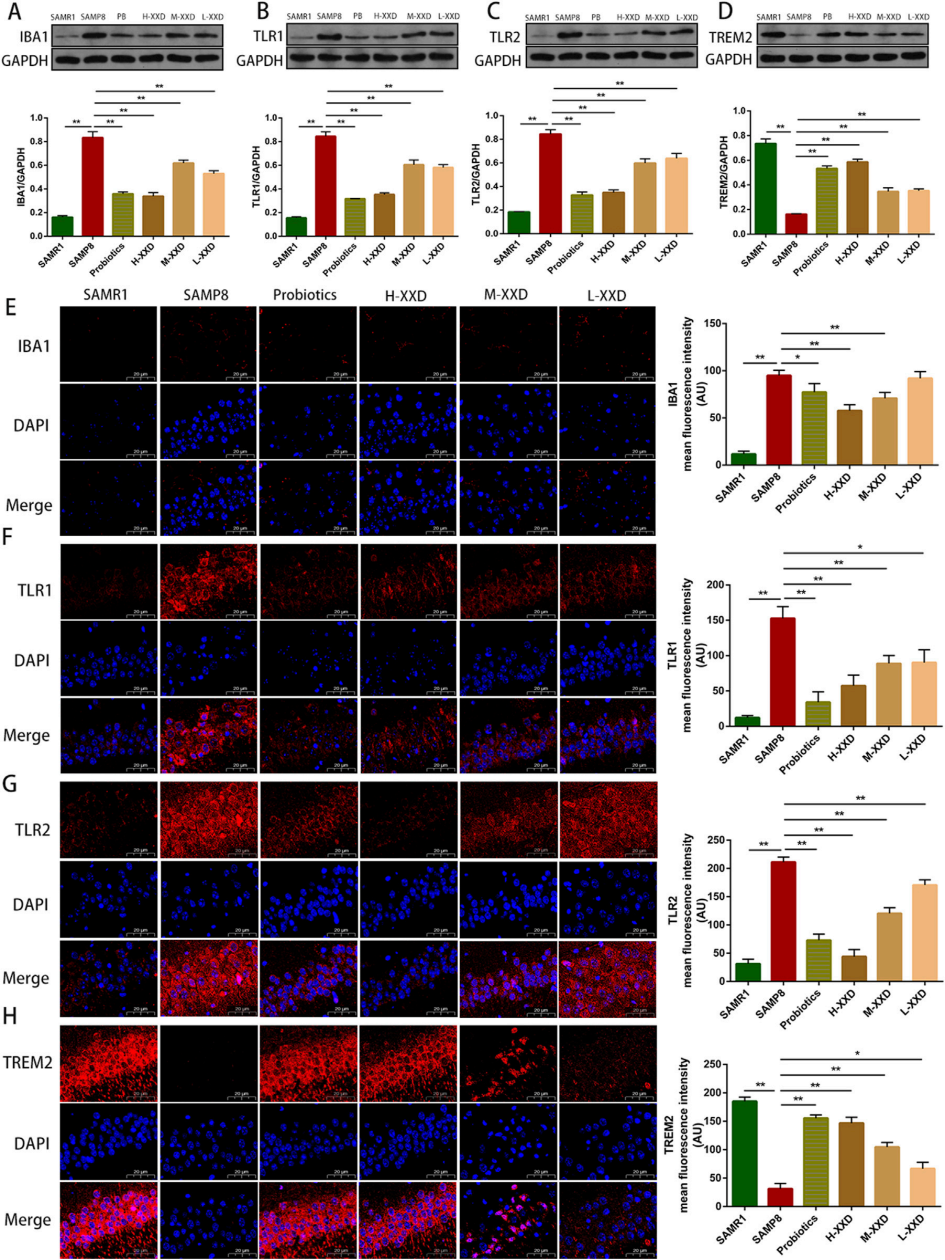
XXD Upregulates TREM2 Expression and Downregulates IBA1, TLR1, and TLR2 Expression, Inhibiting Microglial Activation; **(A)** and **(E)** Analysis of relative IBA1 protein expression levels and average fluorescence intensity of IBA1 in the hippocampal CA1 region of mice; **(B)** and **(F)** Analysis of relative TLR1 protein expression levels and average fluorescence intensity of TLR1 in the hippocampal CA1 region of mice; **(C)** and **(G)** Analysis of relative TLR2 protein expression levels and average fluorescence intensity of TLR2 in the hippocampal CA1 region of mice; **(D)** and **(H)** Analysis of relative TREM2 protein expression levels and average fluorescence intensity of TREM2 in the hippocampal CA1 region of mice; (n = 6); Scale bar: 20μm; images captured at ×400 magnification; **p < 0.01, *p < 0.05.

**FIGURE 9:**
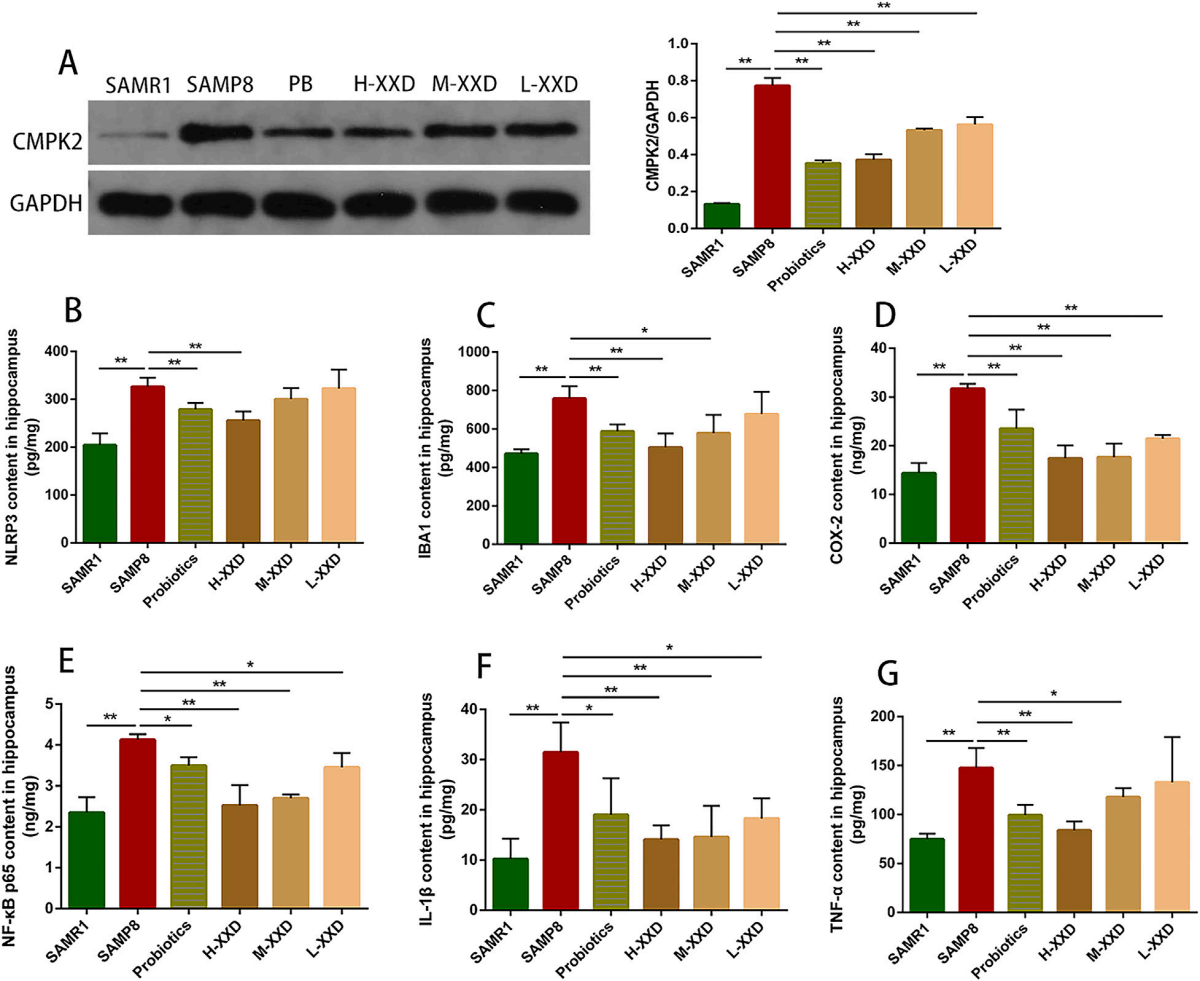
XXD reduces the levels of inflammation-related molecules and cytokines in the hippocampus: **(A)** Comparison of relative expression levels of CMPK2 protein in the hippocampus of different groups of mice; **(B)** Comparison of NLRP3 protein levels in the hippocampus of different groups of mice; **(C)** Comparison of IBA1 protein levels in the hippocampus of different groups of mice; **(D)** Comparison of COX-2 protein levels in the hippocampus of different groups of mice; **(E)** Comparison of NF-κB p65 protein levels in the hippocampus of different groups of mice; **(F)** Comparison of IL-1β protein levels in the hippocampus of different groups of mice; **(G)** Comparison of TNF-α protein levels in the hippocampus of different groups of mice. (n = 6); **p < 0.01, *p < 0.05.

The present research demonstrated that TREM2 expression in the hippocampus of SAMP8 mice was decreased, while IBA1, TLR1, and TLR2 expression significantly increased. Furthermore, the average fluorescence intensity of TMER2 in the mouse hippocampal CA1 region decreased, while the average fluorescence intensities of IBA1, TLR1, and TLR2 increased. Therefore, reduced TMER2 expression in the hippocampal region of SAMP8 mice can upregulate IBA1 expression and promote the overexpression of TLR1 and TLR2, thereby mediating microglia-related neuroinflammation. In addition, CMPK2 protein expression and NLRP3, NF-κB p65, COX-2, TNF-α, and IL-1β levels in hippocampal tissue were significantly increased, suggesting that microglia-related neuroinflammation in the brains of SAMP8 mice may be achieved through the CMPK2/NLRP3 and TLRs/NF-κB pathways. Compared with the SAMP8 group, in all XXD-dose groups, TMER2 expression in the mouse hippocampal region increased (Figure 8D), while IBA1, TLR1, and TLR2 expression significantly decreased (Figures 8A–C). The mean fluorescence intensity of TMER2 in the hippocampal CA1 region increased (Figure 8H), while the average fluorescence intensities of IBA1, TLR1, and TLR2 were reduced (Figures 8E–G). CMPK2 protein expression and NLRP3, NF-κB p65, COX-2, TNF-α, and IL-1β levels in hippocampal tissue were significantly decreased (Figures 9A–G). Thus, XXD may inhibit microglial activation by upregulating TMER2 expression, thereby inhibiting the activation of the CMPK2/NLRP3 and TLRs/NF-κB pathways, ultimately leading to reduced neuroinflammation.

## 4 Discussion

This study investigated the mechanism by which XXD ameliorates cognitive impairment in Alzheimer’s disease (AD). The research revealed that AD pathogenesis is a vicious cycle: BBB dysfunction leads to excessive Aβ accumulation, which triggers neuroinflammatory responses driven by microglial activation. This inflammation further damages the BBB, perpetuating the cycle. XXD may interrupt this harmful cascade by modulating both BBB function and neuroinflammation.

### 4.1 Abnormal expression of BBB transport-related proteins and receptors promotes excessive Aβ accumulation in the AD brain

The blood-brain barrier (BBB) serves as a selective and semi-permeable boundary, restricting the infiltration of pathogens, neurotoxins, immune factors, and macromolecules from the peripheral blood into the central nervous system (CNS) (Tao et al., 2022). Simultaneously, it selectively allows the passage of essential nutrients necessary for maintaining healthy brain function (Jones and Shusta, 2007). There is substantial evidence suggesting that significant BBB dysfunction in Alzheimer’s disease (AD) results in excessive accumulation of β-amyloid (Aβ) (Wang et al., 2021). The excessive deposition of Aβ in AD is a result of an imbalance between its production and clearance. The blood-brain barrier (BBB), a critical structure involved in the clearance of metabolic waste from the brain, includes various transporter proteins such as P-gp, Mfsd2a, RAGE, LRP-1, MRP, CB1, and CB2 (Pyun et al., 2022; Huang and Li, 2021; Stella, 2010; Hillard, 2018; Wang et al., 2018; Ma et al., 2018; Nies et al., 2004), which mediate the transport of Aβ into and out of the brain. *In vitro* studies demonstrated that the LPS-induced BBB damage model exhibited reduced expression of P-gp mRNA and protein, decreased levels of CB2 and Mfsd2a, and increased CB1 protein expression, suggesting aberrations in Aβ transport-related proteins and receptors under BBB damage conditions. To confirm these expression patterns in an *in vivo* AD model, SAMP8 mice were employed. The results showed impaired spatial learning and memory abilities in SAMP8 mice, along with increased Evans blue extravasation in brain tissue, indicating that cognitive deficits are linked to BBB dysfunction. Additionally, a significant rise in Aβ_1-42_ content in the hippocampus of SAMP8 mice was observed, and abnormalities were noted in the P-gp/ECS axis, the RAGE/LRP1 receptor system, as well as MRP2 and Mfsd2a levels, suggesting that the dysregulation of BBB transport-related proteins and receptors in AD contributes to excessive Aβ accumulation.

### 4.2 Aβ pathology promotes microglia-related neuroinflammation in the AD brain

Neuroinflammation, a consistent pathological feature throughout the progression of Alzheimer’s disease (AD), accelerates the onset and progression of the disease. Studies indicate a potential link between Aβ and neuroinflammation in AD, where pathological Aβ deposition is associated with the activation of macrophages, microglia, and astrocytes, as well as the release of pro-inflammatory cytokines (Ying et al., 2023). Microglia are pivotal in driving neuroinflammation in AD; Aβ aggregation induces sustained microglial activation, resulting in elevated secretion of inflammatory cytokines and chemokines that further exacerbate microglial activity (Huang et al., 2022). Thus, alleviating Aβ accumulation in the brain may be essential for mitigating microglia-mediated neuroinflammation in AD. The results of this study show that reduced TREM2 expression in the hippocampus of SAMP8 mice can upregulate IBA1 expression and enhance the overexpression of TLR1 and TLR2, thereby mediating microglia-related neuroinflammation. Further analysis of downstream inflammatory molecules and cytokines (CMPK2, NLRP3, NF-κB p65, COX-2, TNF-α, and IL-1β) indicated that microglia-related neuroinflammation in the brains of SAMP8 mice might be mediated through the CMPK2/NLRP3 and TLRs/NF-κB pathways. Additionally, a significant increase in the Aβ content in the hippocampus of SAMP8 mice was observed, confirming that microglia-related neuroinflammation and Aβ pathology mutually promote each other.

### 4.3 Microglial activation-mediated neuroinflammation exacerbates BBB damage in the AD brain

Microglia are brain-resident cells that play a crucial role in immune responses within the brain. In the AD brain, activated microglia are frequently observed surrounding Aβ plaques and release inflammatory cytokines such as IL-1, IL-6, TNF-α, and TGF-β (da Fonseca et al., 2014). These inflammatory cytokines can downregulate the expression of tight junction proteins and disrupt BBB integrity through both direct and indirect pathways, thereby exacerbating BBB leakage (Hussain et al., 2021). In addition, neuroinflammation also exerts detrimental effects on BBB transport function. Studies have shown that neuroinflammation leads to a significant increase in the levels of P-gp and RAGE proteins in rat brain microvessels, both of which are critical for the bidirectional transport of Aβ across the BBB (Lai et al., 2021).

In rat brain capillaries, TNF-α exposure can induce a series of cascade events that lead to the activation of the transcription factor NF-κB, which regulates the expression and activity of P-gp, breast cancer resistance protein (BCRP), and MRP1 (Chaves et al., 2017), thus disrupting BBB transport function. In another study, the pro-inflammatory cytokine IL-6 resulted in a reduction in the immunostaining area fraction of P-gp, zonula occludens-1 (ZO-1), and aquaporin 4 (AQP4) in the brain microvessels of wild-type mice. Treatment with the anti-inflammatory cytokine IL-10 led to an increase in the immunostaining area fraction of AQP4, providing the first evidence that IL-10 can at least partially prevent IL-6-induced BBB damage (Barabási et al., 2023). In summary, neuroinflammation mediated by microglial activation exacerbates BBB integrity and transport dysfunction. Related studies have shown that LPS promotes the secretion of cytokines and chemokines by microglial cells, which are closely associated with the neuroinflammatory process (He et al., 2021). Consequently, in this *in vitro* study, LPS was employed as an inducer for the BBB inflammation damage model.

The findings demonstrated that LPS reduced P-gp mRNA and protein expression, increased CB1 expression, and decreased CB2 and Mfsd2a expression in the *in vitro* BBB model, indicating that LPS-induced neuroinflammation leads to aberrant expression of the P-gp/ECS axis and Mfsd2a, thereby impairing BBB transport function. Additionally, the *in vivo* study revealed that reduced TREM2 expression in the hippocampal tissue of SAMP8 mice upregulated IBA1 expression and promoted overexpression of TLR1 and TLR2, thus mediating microglia-related neuroinflammation. This neuroinflammation may be mediated through the CMPK2/NLRP3 and TLRs/NF-κB pathways. Further investigation showed that Evans blue extravasation in the brain tissue of SAMP8 mice was significantly elevated, while the expression of the P-gp/ECS axis, the RAGE/LRP1 receptor system, MRP2, and Mfsd2a in hippocampal tissue was markedly abnormal, indicating damage to BBB integrity and transport function. Consequently, microglial activation-mediated neuroinflammation in the AD brain exacerbates BBB integrity and transport function impairments, which, in turn, amplifies neuroinflammation, forming a vicious cycle (Takata et al., 2021).

### 4.4 By modulating the BBB transport function to reduce Aβ accumulation and mitigate neuroinflammation, XXD ameliorates cognitive deficits in AD

This study demonstrates the effectiveness of XXD in mitigating the deleterious cascade reaction involving blood-brain barrier (BBB) transport dysfunction, excessive Aβ accumulation, and neuroinflammation. The results show that XXD can not only regulate and restore BBB integrity and transport function, thereby reducing pathological Aβ accumulation in the brain, but also suppress neuroinflammatory responses induced by Aβ accumulation. *In vitro* results show that XXD drug-containing serum culture for 24, 48, and 72 h can upregulate P-gp mRNA and protein expression, downregulate CB1 protein expression, and upregulate CB2 and Mfsd2a protein expression, indicating that XXD may improve BBB transport dysfunction by regulating the expression of transporter-related proteins Mfsd2a and the P-gp/ECS system. Subsequent *in vivo* studies revealed that XXD may reduce Aβ accumulation in the brains of SAMP8 mice by regulating the P-gp/ECS axis, the RAGE/LRP1 receptor system, and MRP2 and Mfsd2a proteins, promoting Aβ efflux across the BBB and inhibiting Aβ influx across the BBB. Further investigation found that XXD could upregulate TMER2 expression and downregulate IBA1, TLR1, TLR2, CMPK2 expression, thereby reducing the levels of pro-inflammatory factors NLRP3, NF-κB p65, COX-2, TNF-α, and IL-1β in hippocampal tissue. This suggests that XXD may inhibit microglial activation by upregulating TMER2 expression, thereby inhibiting the activation of the CMPK2/NLRP3 pathway and the TLRs/NF-κB pathway, alleviating neuroinflammation, and improving spatial learning and memory impairments in SAMP8 mice.

Currently, the majority of pharmacological interventions for Alzheimer’s disease (AD) target single pathways. However, due to the complex pathophysiology of AD, monotherapeutic approaches often fall short in achieving comprehensive symptom improvement. Consequently, single-target therapies exhibit limitations in both long-term efficacy and disease-modifying potential. In contrast, XXD demonstrates a distinctive advantage by generating multi-target synergistic effects. Research indicates that XXD can modulate blood-brain barrier (BBB) transport function through the regulation of the P-gp/ECS axis, the RAGE/LRP1 receptor system, and the expression profiles of MRP2 and Mfsd2a proteins. This modulation facilitates the efflux of Aβ_1-42_ and reduces Aβ deposition within the cerebral parenchyma. As a result, the activation of microglia triggered by Aβ oligomerization is attenuated, and the activation of the CMPK2/NLRP3 and TLRs/NF-κB pathways is suppressed. This leads to a reduction in the synthesis of pro-inflammatory cytokines and chemokines, alleviating microglia-mediated neuroinflammation in the AD brain and ultimately reducing AD-associated cognitive deficits. This multi-faceted mechanism not only enhances therapeutic efficacy but also potentially mitigates the risk of developing resistance, which is commonly associated with single-target pharmacological interventions.

Although this study has initially elucidated the multi-target mechanism of XXD, the intricate interaction networks and precise regulatory pathways require further investigation. Future research could employ cutting-edge methodologies, such as high-throughput screening, proteomic analysis, and metabolomic profiling, to further elucidate the complex mechanisms underlying XXD’s multi-target actions. Such investigations are crucial for unveiling the comprehensive, multi-tiered regulatory framework that underpins XXD’s therapeutic efficacy in AD management.

## Data Availability

The original contributions presented in the study are included in the article/supplementary material, further inquiries can be directed to the corresponding author.
